# Intra-operative physiologic interventions associated with postoperative neurological complications in neonates and infants: A secondary analysis of the NECTARINE cohort

**DOI:** 10.1097/EJA.0000000000002308

**Published:** 2026-02-02

**Authors:** Virginia E. Tangel, Sanne E. Hoeks, Laszlo Vutskits, Robert Jan Stolker, Nicola Disma, Jurgen C. de Graaff, on behalf of the NECTARINE Group of the European Society of Anaesthesiology and Intensive Care Clinical Trial Network, Christian Breschan

**Affiliations:** From the Department of Anaesthesiology, Erasmus MC, Rotterdam, The Netherlands (VET, SEH, RJS, JCdG), Department of Anaesthesiology, Weill Cornell Medicine, New York, New York, USA (VET, JCdG), Division of Anaesthesiology, Department of Acute Care Medicine, University Hospitals of Geneva (LV), Geneva Neuroscience Centre, University of Geneva, Geneva, Switzerland (LV), and Unit for Research in Anaesthesia, IRCCS Istituto Giannina Gaslini, Genoa, Italy (ND)

## Abstract

**BACKGROUND:**

Neonates and infants are among the most vulnerable populations to undergo procedures necessitating general anaesthesia and are at risk for postoperative neurological complications. The objective of this study was to assess the association between interventions for intra-operative physiologic instability and the development of postoperative neurological complications in a cohort of neonates and infants undergoing operative and nonoperative procedures.

**METHODS:**

Data for this study were extracted from the NECTARINE study: a prospectively collected cohort of neonates and infants receiving anaesthesia for operative and nonoperative procedures from 165 participating hospitals in 31 countries.

In a multivariable logistic regression model predicting the development of a neurologic complication up to 30 days postoperatively, exposures of interest were binary indicators of unexpected intra-operative instability of systemic homeostasis necessitating a corrective action (based on blood pressure, heart rate, end-tidal and/or blood CO_2_, glucose and sodium).

**RESULTS:**

One hundred and forty out of 5504 (2.5%) neonates and infants developed a postoperative neurologic complication. No statistically significant association was found between interventions for intra-operative physiologic instability and the development of a postoperative neurologic complication.

**CONCLUSION:**

In this secondary analysis of neonates and infants undergoing cardiac and noncardiac procedures, we found a higher rate of neurologic complications compared to adult populations undergoing noncardiac surgery. There was no association between unexpected intra-operative instability of systemic homeostasis necessitating a corrective action and subsequent neurologic complications; however, these findings do not necessarily indicate the absence of any effect of physiological disturbances on neurologic outcomes.

## Graphical abstract

**Figure:**
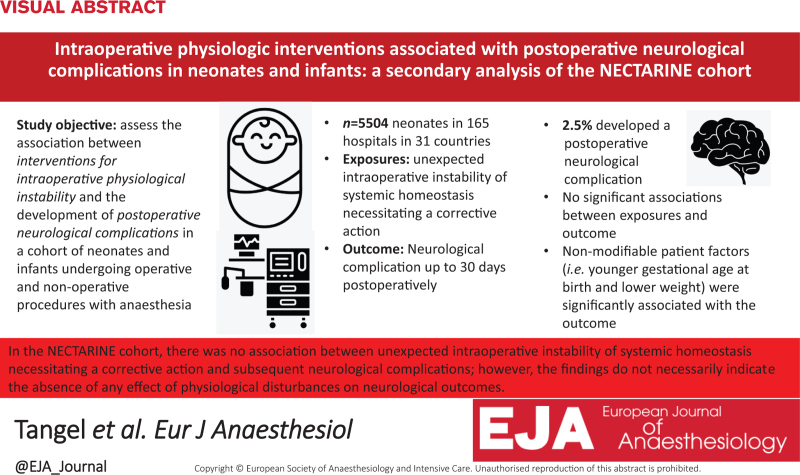

**KEY POINTS**

- This study examines the association between interventions to correct for intra-operative instability and neurologic complications in a cohort of neonates and infants from 31 countries.
- In neonates and infants, it is unknown whether and how peri-operative neurological complications are associated with intra-operative disturbances of physiological homeostasis.
- The analysis describes an overall incidence of 2.5% of neurological complications in neonates and infants following anaesthesia and surgery.
- There was no statistically significant association between the occurrence of interventions aimed to correct homeostasis and neurological complications.

## Introduction

Neonates and infants are among the most vulnerable populations to undergo procedures necessitating general anaesthesia and are at risk for severe postoperative neurologic complications.^1–4^ The aetiology of postoperative neurological complications is not entirely understood. Disturbances of systemic homeostasis such as decreased cardiac output, hypotension and acid-base disturbances have been proposed as major contributors due to their potential effects on cerebral perfusion.^5,6^ Whether and how physiologic instability during the peri-operative period in neonates is associated with adverse neurological outcomes has not been previously explored. In a recent prospective observational multicentre study (NECTARINE), we identified thresholds of predetermined physiologic variables that triggered corrective medical interventions during the peri-operative course in 5609 neonates and infants.^7^ There is no consensus for what constitutes ‘normal’ intra-operative physiologic parameters in paediatric anaesthesia, nor are there universally accepted thresholds for treatment; describing these patterns was one goal of the NECTARINE study. Accordingly, there were no specified physiological thresholds which would necessitate treatment nor directives regarding the choice of treatments in the study; practice patterns were allowed to vary by centre and individual anaesthesiologist.

In the NECTARINE population, the incidence of predetermined critical events requiring intervention was 35.3% (95% CI: 34.1 to 36.4), but there was marked heterogeneity in terms of the range of physiological parameters that triggered interventions within age categories. Critical events occurred more often in very young patients (infants under 32 weeks postmenstrual age at the time of surgery). Risk factors associated with critical events included pre-operative intensive support, neonatal medical conditions (i.e. presence of cardiovascular or respiratory support or admission from ICU), and comorbidities present at the time of surgery. Episodes of cardiovascular instability (in terms of both blood pressure and heart rate) accounted for 60.7% of all interventions.

The objective of the present secondary analysis was to assess the association between interventions for intra-operative physiological instability and the development of postoperative neurological complications in the NECTARINE cohort of neonates and infants undergoing operative and nonoperative procedures. Specifically, we hypothesised that the development of postoperative neurological complications in the NECTARINE study population was independently associated with unexpected instability of systemic homeostasis necessitating a corrective action.

## Materials and methods

Data for this retrospective secondary analysis were extracted from the NECTARINE study: a multicentre, prospective cohort of neonates and infants up to 60 weeks postmenstrual age collected between 1 March 2016 and 31 January 2017. Results from the main analyses have been previously published,^7^ and the study protocol was registered at ClinicalTrials.gov (NCT02350348). This secondary analysis was registered *a priori* with the Open Science Foundation: https://osf.io/b5j3g/. STROBE guidelines were followed in reporting.^8^

For the original study, all 165 participating centres in 31 countries who contributed cases to the database obtained local ethical approval for data collection. The study protocol covering data collection and all subsequent analyses was approved at IRCCS AOU San Martino – IST (374REG2015, PI: Prof. Francesco De Stefano, approval date: 13 October 2015). The NECTARINE study is at the level of the anaesthesia case, meaning that multiple procedures could have occurred within the same anaesthetic, and an individual patient could be included multiple times if undergoing multiple separate procedures with anaesthesia during the data collection period.

### Primary outcome

The primary outcome was a binary composite measure indicating the development of any neurological complication up to 30 days postoperatively; the primary outcome was ascertained at a 30-day follow up *via* face-to-face interview, through medical records (if the patient was still hospitalised), or *via* a standardised telephone interview with the parent/caregiver if the patient had been discharged. Neurological complications included hypertonia (new onset), hypotonia (new onset), intracranial bleeding (ultrasound, CT or MRI), intracranial ischaemia (ultrasound, CT or MRI) and/or occurrence of seizures (clinically or EEG).

### Secondary outcomes

Secondary outcomes were the neurological complications comprising the primary outcome, divided into outcomes ascertained either *via* clinical observation or *via* imaging (ultrasound, CT or MRI). The manner of identification of a neurological complication (by clinical observation or radiological imaging) was not mutually exclusive within a case, and multiple different types of neurological complications could be identified within the same case. Eleven out of 6542 cases in the database were marked as having a postoperative neurological complication but were omitted from the present analysis because data from the original case report form (CRF) did not contain the requisite additional detail on the nature of the neurologic complication.

### Covariates

Exposures of interest were binary indicators of unexpected intra-operative instability of systemic homeostasis necessitating a corrective action in terms of cardiac output (either blood pressure or heart rate, measured separately), CO_2_, glucose and sodium. The physiologic indicators of instability were based on questions in the NECTARINE CRF (Supplemental Digital Content 1). An intervention was coded as present if it occurred in any of the patient's anaesthesia cases. Although the NECTARINE data were recorded at the anaesthesia case level, we conducted our analyses at the level of the patient, as follow-up was only conducted once per patient, 30 days from the last anaesthesia exposure.

To account for potential confounding introduced by procedural risk, we retrospectively classified each procedure into rank-ordered categories based on the complexity of the anaesthesia for the procedure (Supplemental Digital Content 2); in the case of a patient who had multiple procedures, this covariate was coded to reflect the anaesthesia procedure with the greatest risk. Gestational age at birth (weeks), chronological age (weeks), weight at birth (kg), weight at index procedure (kg), total anaesthesia duration across all anaesthesia cases (min), anaesthesia procedural complexity (four categories), number of total anaesthesia exposures and binary variables to identify the presence of a cardiac procedure and neurological procedure served as confounders in multivariable models. An additional 148 out of 6542 cases were dropped due to missing data on total anaesthesia time, height or weight; or the cases were unable to be classified into a category of anaesthesia procedural complexity. In total, omitting these cases resulted in the attrition of less than 2% of patients from the present analysis.

### Statistical analyses

Descriptive statistics were calculated at the level of the patient by category of the primary outcome to characterise the study cohort, and all variables were compared using χ^2^ or *t*-tests (or nonparametric equivalents, where appropriate). Multivariable logistic regression models for each outcome were fitted with all exposure variables and confounders as covariates using complete case analysis. Adjusted ORs (with 95% confidence intervals) are presented, and *P* values were adjusted *posthoc* to account for multiple testing. Cohort building and analyses were conducted in Stata 16 SE (StataCorp, College Station, Texas, USA). Significance was evaluated at α = 0.05.

## Results

One hundred and forty out of 5504 (2.5%) neonates and infants developed a postoperative neurological complication up to 30 days postoperatively; 57 (1.0%) of these patients experienced seizures, 46 (0.8%) had documented intracranial bleeding, 24 (0.4%) had documented intracranial ischaemia, 20 (0.4%) developed hypotonia (new onset) and 13 (0.2%) developed hypertonia (new onset). Eighty-two out of 5504 (1.5%) neonates experienced a postoperative neurological complication which was identified *via* clinical observation. Sixty-seven out of 5504 (1.2%) experienced a postoperative neurological complication which was identified *via* imaging; complications could be identified in more than one modality, and multiple forms of neurological complications could occur per patient.

In bivariate analyses, a higher percentage of neonates with postoperative neurological complications experienced intra-operative disturbances to blood pressure (40.7 vs. 21.1%), CO_2_ (16.4 vs. 8.7%) and heart rate (7.9 vs. 2.3%), which required intervention compared to neonates without neurologic complications (Table 1). Neurological complications were also more likely to develop in patients born at a younger gestational age, at a lower chronological age at the time of the index procedure, at a smaller weight at the time of the index procedure, and in more complex and numerous procedures. However, after correction for confounding, no exposures related to interventions for unexpected instability of systemic homeostasis were significantly associated with the development of a postoperative neurologic complication (Table 2).

**Table 1:** Descriptive statistics of cohort by category of primary outcome

	Total	*n* (%) among cases without neurological complication(s)	*n* (%) among cases with neurological complication(s)	*P*
				
Intervention for:				
Hypocapnia or hypercapnia	487 (8.9%)	464 (8.7%)	23 (16.4%)	0.001
Hypotension	1189 (21.6%)	1132 (21.1%)	57 (40.7%)	<0.001
Bradycardia or tachycardia	133 (2.4%)	122 (2.3%)	11 (7.9%)	<0.001
Hyperglycaemia	54 (1.0%)	51 (1.0%)	3 (2.1%)	0.16
Hypoglycaemia	108 (2.0%)	104 (1.9%)	4 (2.9%)	0.43
Hypernatraemia	9 (0.2%)	8 (0.2%)	1 (0.7%)	0.10
Hyponatraemia	16 (0.3%)	14 (0.3%)	2 (1.4%)	0.01
Patient demographics				
Gestational age (weeks)	38 [35 to 39]	38 [35 to 39]	33 [27 to 38]	<0.001
Chronological age at index procedure (weeks)	8 [3 to 13.7]	8.14 [3.14 to 13.86]	3.21 [1.29 to 10.14]	<0.001
Weight at birth (kg)	2.96 [2.12 to 3.44]	2.98 [2.18 to 3.45]	1.95 [1.02 to 2.93]	<0.001
Weight at index procedure (kg)	4 [3.07 to 5.13]	4 [3.1 to 5.17]	2.5 [1.6 to 3.53]	<0.001
Procedural characteristics				
Total anaesthesia time (min)	95 [60 to 174]	92 [60 to 170]	142.5 [85.0 to 246.0]	<0.001
Risk categories (reference: first/lowest risk)	2081 (37.8)	2068 (38.6)	13 (9.3)	<0.001
Mild risk	747 (13.6)	729 (13.6)	18 (12.9)	
Moderate risk	2154 (39.1)	2073 (38.7)	81 (57.9)	
Highest risk	522 (9.5)	494 (9.2)	28 (20.0)	
Cardiac procedure	386 (7.0)	364 (6.8)	22 (15.7)	<0.001
Neuro procedure	271 (4.9)	244 (4.6)	27 (19.3)	<0.001
Number of total anaesthesia procedures	1 [1 to 1]	1 [1 to 1]	1 [1 to 2]	<0.001
*n*	5504	5364	140	

Data presented as *n* (percentage) or median [interquartile range].

**Table 2:** Unadjusted and adjusted odds ratios for primary outcome

	Unadjusted odds ratio (OR)	95% confidence interval (CI)	Adjusted odds ratio (aOR)	95% confidence interval (CI)	*P*	Bonferroni corrected *P*
						
Intervention for:						
Hypocapnia or hypercapnia	2.08	(1.31 to 3.28)	1.00	(0.60 to 1.67)	0.990	1.000
Hypotension	2.57	(1.82 to 3.62)	1.33	(0.89 to 1.99)	0.160	0.791
Bradycardia or tachycardia	3.66	(1.93 to 6.96)	1.70	(0.83 to 3.51)	0.149	1.000
Hyperglycaemia	2.28	(0.70 to 7.40)	1.02	(0.28 to 3.73)	0.972	1.000
Hypoglycaemia	1.49	(0.54 to 4.10)	0.63	(0.21 to 1.83)	0.394	1.000
Hypernatraemia	4.82	(0.60 to 38.77)	2.43	(0.22 to 26.82)	0.470	1.000
Hyponatraemia	5.54	(1.25 to 24.60)	1.23	(0.18 to 8.32)	0.832	1.000
Patient demographics						
Gestational age (weeks)	0.87	(0.85 to 0.90)	0.91	(0.84 to 0.99)	0.026	
Chronological age at index procedure (weeks)	0.94	(0.92 to 0.97)	1.01	(0.97 to 1.05)	0.672	
Weight at birth (kg)	0.52	(0.44 to 0.61)	1.32	(0.83 to 2.10)	0.240	
Weight at index procedure (kg)	0.47	(0.41 to 0.53)	0.56	(0.43 to 0.73)	0.000	
Procedural characteristics						
Total anaesthesia time (min)	1.00	(1.00 to 1.00)	1.00	(1.00 to 1.00)	0.785	
Risk categories (reference: first/lowest risk)						
Mild risk	3.93	(1.92 to 8.06)	3.66	(1.77 to 7.60)	0.000	
Moderate risk	6.22	(3.45 to 11.20)	2.70	(1.42 to 5.13)	0.003	
Highest risk	9.02	(4.64 to 17.53)	3.82	(1.39 to 10.50)	0.009	
Cardiac procedure	2.56	(1.60 to 4.09)	1.03	(0.41 to 2.61)	0.947	
Neuro procedure	5.01	(3.23 to 7.78)	3.63	(2.19 to 6.00)	0.000	
Number of total anaesthesia procedures	1.62	(1.38 to 1.90)	1.26	(1.01 to 1.58)	0.043	
*n*	5504		5504			

In multivariable analyses of the secondary outcomes, an intervention to correct for intra-operative disturbances to heart rate (either bradycardia or tachycardia) was associated with an increased risk of developing a postoperative neurological complication identified *via* clinical observation up to 30 days postoperatively (adjusted odds ratio: 3.17, 95% CI: 1.38 to 7.26, Bonferroni-adjusted *P* *=* 0.04, Table 3). No exposures related to interventions for unexpected instability of systemic homeostasis were associated with a neurological complication ascertained from imaging.

**Table 3:** Adjusted odds ratios for secondary exploratory outcomes

	Neurological outcome: clinically ascertained (*n* = 82)				Neurological outcome: ascertained *via* imaging (*n* = 67)			
		
	*n* (% among cases with complications)	Adjusted odds ratio (aOR)	95% confidence interval (CI)	*P*	*n* (% among cases with complications)	Adjusted odds ratio (aOR)	95% confidence interval (CI)	*P*
								
Intervention for:								
Hypocapnia or hypercapnia	12 (14.6%)	1.01	(0.51 to 2.00)	0.985	13 (19.4%)	1.15	(0.58 to 2.29)	0.691
Hypotension	24 (29.3%)	0.85	(0.49 to 1.48)	0.574	36 (53.7%)	2.04	(1.15 to 3.62)	0.014
Bradycardia or tachycardia	8 (9.8%)	3.17	(1.38 to 7.26)	0.006	4 (6.0%)	0.90	(0.27 to 2.95)	0.861
Hyperglycaemia	2 (2.4%)	1.00	(0.14 to 7.10)	0.999	1 (1.5%)	0.70	(0.08 to 5.78)	0.737
Hypoglycaemia	1 (1.2%)	0.33	(0.04 to 2.55)	0.289	3 (4.5%)	0.85	(0.23 to 3.08)	0.804
Hypernatraemia	1 (1.2%)	11.15	(0.61 to 203.02)	0.103	0 (0.0%)	–	–	–
Hyponatraemia	0 (0.0%)	–	–		2 (3.0%)	2.99	(0.42 to 21.13)	0.273
*N*	82	5488			67	5495		

^a^*n* = 16 observations were dropped from the multivariable model for clinically ascertained neurological complications due to a lack of cases with hyponatraemia; *n* = 9 observations were dropped from the multivariable model for neurological complications ascertained via imaging due to a lack of cases with hypernatraemia. Both models controlled for patient and procedural characteristics.

## Discussion

We report a 2.5% incidence of postoperative neurologic complications after anaesthesia in a multicentre cohort of more than 5000 neonates and infants from 165 hospitals across Europe. The incidence of these complications in this very young population is higher than reported in an adult population following noncardiac surgery (0.5%)^9^ and is comparable to the incidence observed in adult patients following mitral valve replacement.^10^ Given the paucity of available data on peri-operative neurological complications in neonates and infants, our study provides important new insights about the incidence of these morbidities. Previous information on neurological complications in these populations stems primarily from case series describing neuromorbidity following anaesthesia and surgery in young children.^5^ Although these reports raised awareness about the possibility of peri-operative neurological injury, the question of how frequent these events occur remained unexplored. The prospective multicentric design of the NECTARINE study with a detailed follow-up was appropriately designed to investigate this issue.

We found that instability in physiological parameters severe or prolonged enough to necessitate an intervention was not associated with a neurological complication up to 30 days postoperatively. These observations are in line with an earlier study which found no association between intra-operative hypotension and long-term adverse neurodevelopmental outcomes in children exposed to multiple anaesthetics prior to age 3 years.^11^ The only factors that significantly increased the odds of a postoperative neurologic complication in the present study were younger gestational age, lower weight at the time of the index procedure, greater procedural risk, undergoing a neurological procedure and a greater total number of exposures to anaesthesia (Table 2); however, these patient and procedural factors were not tested as part of our hypothesis, and thus, we are unable to draw conclusions about these findings.

Some research in adult patients showed that peri-operative blood pressure is related the development of postoperative neurologic complications, including ischaemic stroke,^12^ though other studies have not established an association.^9,13^ However, the importance of most studies on this topic is limited because they are retrospective and not randomised trials.^14^

## Limitations

Although we did find a significant association of an intervention to correct for intra-operative heart rate instability and the secondary outcome of a development of a clinically ascertained postoperative neurological outcome, we are limited by the precision of the exposure measure to make inferences about this finding. Because the NECTARINE database did not capture physiological data directly from the electronic medical record, the true incidence of physiologic instability may be undercounted (e.g. in cases wherein brief instability was present but was not prolonged enough to necessitate treatment). Moreover, results from multivariable models of our secondary outcomes are exploratory due to the low number of events per variable.

Although this study did not establish a relationship between intra-operative instability and the development of a postoperative neurological complication, our findings should not be interpreted to suggest that intra-operative disturbances to systemic homeostasis can or should be ignored by the anaesthesia care team, nor is this an invitation to delay an intervention. Using interventions to correct for instability as predictor variables for neurological complications (as we have in this study) is not the same as quantifying the physiological instability itself; this imprecision in measurement is perhaps the biggest shortcoming of the present analysis. Employing advanced techniques to quantify cerebral blood flow velocity would be necessary to more precisely capture instances of changes to cerebral perfusion beyond standard monitoring.^15^ Only 8% of the NECTARINE cohort received NIRS monitoring, which we judged was too small of a sample to conduct a sensitivity analysis in this population.

Importantly, there are multiple other potential causal factors related to the onset of neurological complications following anaesthesia and surgery in neonates other than intra-operative disturbances to systemic homeostasis; due to data limitations, we were only able to control for certain patient, procedural and intra-operative characteristics.

The primary aim of the original NECTARINE study was to identify physiological thresholds that triggered interventions for critical events, and not necessarily the direction of the instability which required intervention. Thus, it was not feasible to distinguish if a patient experienced hypocapnia or hypercapnia which required intervention (nor bradycardia or tachycardia) from the available data. For that reason, some of our exposures are limited to a binary variable which measures only if an intervention was required, but neither the direction nor duration of the instability that predicated the intervention. However, upon examination of the data, it was evident that all interventions to correct blood pressure were due to hypotension (Supplemental Digital Content 3).

Notably, the instances of documented hyperglycaemia, hypoglycaemia, hypernatraemia and hyponatraemia were much lower in this database than hypocapnia or hypercapnia, hypotension, and bradycardia or tachycardia. Blood pressure, heart rate and oxygen are continuously measured as part of standard of care in the intra-operative period. Instances of hypo/hyperglycaemia and hypo/hypernatraemia would not be measured continuously (or measured at all bereft of an indication), and identification and treatment would be reliant on vigilance of the treating physician. Thus, it is possible that the true incidence of some instances of systemic instability that would have predicated an intervention are undercounted.

Across the 165 participating centres, there was considerable heterogeneity in practice patterns. This heterogeneity stems from the fact that there are no specific standards for physiological cut-off values triggering action in neonatal populations (or, in some instances, these cut-off values are highly debated, which reflects heterogeneity in practice). We believe that this heterogeneity could facilitate the detection of independent associations between physiological values and neurological complications, provided the existence of an adequate sample size. The fact that we could not detect any association does not necessarily mean the absence of effects of physiological variables on neurological outcomes because there is a biological plausibility. It merely means that despite our large cohort, we were not able to detect such associations.

Prior to the NECTARINE study, there were no other large databases that captured intra-operative data about neonates and infants undergoing anaesthesia and which also contained additional information on outcomes. Therefore, despite the obvious limitations, we believe that at present the NECTARINE database is the only database uniquely suited to answering this research question.

Finally, the composite outcome variable included hypertonia (new onset) and hypotonia (new onset) as neurological complications, as originally specified in the CRF. Although these may not be considered neurological complications themselves, hypertonia or hypotonia can occur as neurological signs or manifestations of underlying complications following surgery in the critically ill neonate or infant. The aetiology of these underlying complications includes infection/sepsis, haemodynamic instability leading to hypoxia or ischaemia, etc.

## Conclusion

In this secondary analysis of neonates and infants having anaesthesia for operative and nonoperative procedures, we found a high rate of neurological complications compared to an adult population undergoing noncardiac surgery. However, we found no association between unexpected intra-operative instability of systemic homeostasis necessitating a corrective action and subsequent neurological complications. These findings should be interpreted with caution, as they do not indicate an absence of any effect of physiological instability on neurological outcomes.

## Supplementary Material

Supplemental Digital Content

## Supplementary Material

Supplemental Digital Content

## References

[1] McCann ME Lee JK Inder T. Beyond anesthesia toxicity: anesthetic considerations to lessen the risk of neonatal neurological injury. *Anesth Analg* 2019; 129:1354–1364. 31517675 10.1213/ANE.0000000000004271 PMC7032045

[2] López SPL Ojeda AZ Gómez GJ Fernández IB. Monitoring of blood pressure is not enough to avoid neonatal postoperative encephalopathy. *Am J Perinatol Rep* 2018; 8:e192–e194. 10.1055/s-0038-1668565 PMC6147745 30250759

[3] Nelson O Wu L Berger JA. A retrospective observational study of post-induction low systolic blood pressure and associated patient and perioperative factors in infants undergoing general anesthesia for inguinal hernia repair. *Anesth Res* 2024; 1:80–90.

[4] Rhee CJ da Costa CS Austin T. Neonatal cerebrovascular autoregulation. *Pediatr Res* 2018; 84:602–610. 30196311 10.1038/s41390-018-0141-6 PMC6422675

[5] McCann ME Schouten A Dobija N. Infantile postoperative encephalopathy: perioperative factors as a cause for concern. *Pediatrics* 2014; 133:e751–e757. 24515520 10.1542/peds.2012-0973

[6] Fedriga M Martini S Iodice FG. Cerebral autoregulation in pediatric and neonatal intensive care: a scoping review. *J Cereb Blood Flow Metab* 2024; 44:1208–1226. 10.1177/0271678X241261944 PMC11542144 38867574

[7] Disma N Veyckemans F Virag K. Morbidity and mortality after anaesthesia in early life: results of the European prospective multicentre observational study, neonate and children audit of anaesthesia practice in Europe (NECTARINE). *Br J Anaesth* 2021; 126:1157–1172. 33812668 10.1016/j.bja.2021.02.016

[8] Von Elm E Altman DG Egger M. The Strengthening the Reporting of Observational Studies in Epidemiology (STROBE) statement: guidelines for reporting observational studies. *J Clin Epidemiol* 2007; 61:344–349. 10.1016/j.jclinepi.2007.11.008 18313558

[9] Marcucci M Painter TW Conen D. Hypotension-avoidance versus hypertension-avoidance strategies in noncardiac surgery: an international randomized controlled trial. *Ann Intern Med* 2023; 176:605–614. 37094336 10.7326/M22-3157

[10] D’Agostino RS Jacobs JP Badhwar V. The Society of Thoracic Surgeons Adult Cardiac Surgery Database: 2019 update on outcomes and quality. *Ann Thorac Surg* 2019; 107:24–32. 30423335 10.1016/j.athoracsur.2018.10.004

[11] Gleich SJ Shi Y Flick R. Hypotension and adverse neurodevelopmental outcomes among children with multiple exposures to general anesthesia: subanalysis of the Mayo Anesthesia Safety in Kids (MASK) Study. *Pediatr Anesth* 2021; 31:282–289. 10.1111/pan.14106 PMC8237208 33320392

[12] Bijker JB Persoon S Peelen LM. Intraoperative hypotension and perioperative ischemic stroke after general surgery: a nested case-control study. *Anesthesiology* 2013; 57:144–145. 10.1097/ALN.0b013e3182472320 22277949

[13] Hsieh JK Dalton JE Yang D. The association between mild intraoperative hypotension and stroke in general surgery patients. *Anesth Analg* 2016; 123:933–939. 27636576 10.1213/ANE.0000000000001526

[14] Yu Q Qi J Wang Y. Intraoperative hypotension and neurological outcomes. *Curr Opin Anesthesiol* 2020; 33:646–650. 10.1097/ACO.0000000000000904 32769747

[15] Rondagh M Kortenbout AJ de Munck S. A comparison of ultrafast and conventional spectral Doppler ultrasound to measure cerebral blood flow velocity during inguinal hernia repair in infants. *J Clin Anesth* 2024; 92:111312. 37926064 10.1016/j.jclinane.2023.111312

