# Supplementary material for: Intra-operative physiologic interventions associated with postoperative neurological complications in neonates and infants: A secondary analysis of the NECTARINE cohort
Source: Eur J Anaesthesiol. 2026 Feb 2;43(4):349–56. doi: 10.1097/EJA.0000000000002308 (PMC12955970; doi:10.1097/EJA.0000000000002308)

**Supplementary Material**

[Supplementary Material 1. Coding of exposure variables. 2](#_Toc190149243)

[Supplementary Material 2: Classification of anaesthesia procedural complexity. 3](#_Toc190149244)

[Supplementary Material 3. Interventions to correct for cardiovascular instability based on blood pressure. 4](#_Toc190149245)

# Supplementary Material 1. Coding of exposure variables.

| Exposure variable | Question(s) in CRF used to define exposure | Limitation | Impact to inference |
| --- | --- | --- | --- |
| Hypocapnia/hypercapnia | Has altered CO2, which needed intervention(s), occurred?  [Yes/No] | Unable to determine direction of instability based on fluids and/or medications given to correct oxygen instability | Limited precision of exposure |
| Hypotension | Has CARDIOVASCULAR INSTABILITY, which needed intervention(s), occurred? Was the intervention/treatment based on blood pressure?  [Yes/No] | After examining the data, we presumed that all interventions for BP were due to hypotension based on the fluids and/or medications administered to correct the instability | Very likely correct, because the interventions identified are only used to treat hypotension (not hypertension) |
| Tachycardia/bradycardia | Has CARDIOVASCULAR INSTABILITY, which needed intervention(s), occurred? Was the intervention/treatment based on ECG disturbance  [Yes/No] | Unable to determine direction of instability based on fluids and/or medications given to correct heart rate instability | Limited precision of exposure |
| Hyperglycemia | Have you performed treatment(s) for high/low glucose and/or Na+? Specify intervention(s) for high/low glucose:  [Stop IV glucose or fluids containing glucose] | None; able to determine if hyperglycaemia was present based on intervention | Correct |
| Hypoglycemia | Have you performed treatment(s) for high/low glucose and/or Na+? Specify intervention(s) for high/low glucose:  [Administration of glucose (IV or continuous infusion)] | None; able to determine if hypoglycaemia was present based on intervention | Correct |
| Hypernatremia | Have you performed treatment(s) for high/low glucose and/or Na+? Specify intervention(s) for high/low Na+:  [Stop IV glucose or hypotonic solutions] | None; able to determine if hypernatremia was present based on intervention | Correct |
| Hyponatremia | Have you performed treatment(s) for high/low glucose and/or Na+? Specify intervention(s) for high/low Na+:  [Administration of additional Na+ (fluids or electrolytes)] | None; able to determine if hyponatremia was present based on answer to intervention | Correct |

# Supplementary Material 2: Classification of anaesthesia procedural complexity.

The first (lowest-risk) category included procedures on external genitalia, the abdominal wall, simple plastic surgery, orthopaedic procedures (non-hip), abscess drainage, and non-surgical procedures under anaesthesia. The second category (mild risk) included surgeries on the hip, clavicle, scapula, or humerus, the Achilles tendon, and the ear (paracentesis, tympanostomy tubes, trans-tympanic drainage). Other procedures in the second category included minimally invasive spinal surgery, facial plastic surgery, ophthalmologic surgeries, and appendectomies via scope. The third category (moderate risk) included visceral surgeries via laparoscopy or laparotomy (except appendectomies and parietal hernias), lung resection (wedge resection, segmentectomy, or lobectomy), pelvic surgery, spinal surgery (< 5 levels), craniotomy, thoracoscopy, bronchoscopy, tonsillectomy, adenoidectomy, rhinoplasty, septoplasty, sinus surgery, labial frenulum correction, dental surgery, esophagoscopy, gastroscopy, and enteroscopy. The fourth (highest-risk) category included heart and major vessels surgery, extended liver resection, transplantation of the liver or kidney, invasive spinal surgery (>6 levels), pneumonectomy, and craniotomy for epilepsy surgery, tumours, vascular malformations, or craniosynostosis.

# Supplementary Table 3. Interventions to correct for cardiovascular instability based on blood pressure.

- - Intervention for cardiovascular instability based on BP (raw case-level data): N=1,315

| Intervention(s) based on BP instability: | Yes (% of total *n*=1315) |
| --- | --- |
| Bolus of > 20 ml kg^-1^ of crystalloids (also 2 x 10 ml kg^-1^) | 732 (55.7%) |
| Bolus of > 10 ml kg^-1^ of albumin | 201 (15.3%) |
| Bolus of > 10 ml kg^-1^ of other colloids | 88 (6.7%) |
| Administration of Fresh Frozen Plasma for hypovolaemia leading to cardiovascular instability | 144 (11.0%) |
| Administration of Packed Red Cells for cardiovascular instability | 218 (16.6%) |
| Other | 219 (16.7%) |

**Other: Specify (free text):**


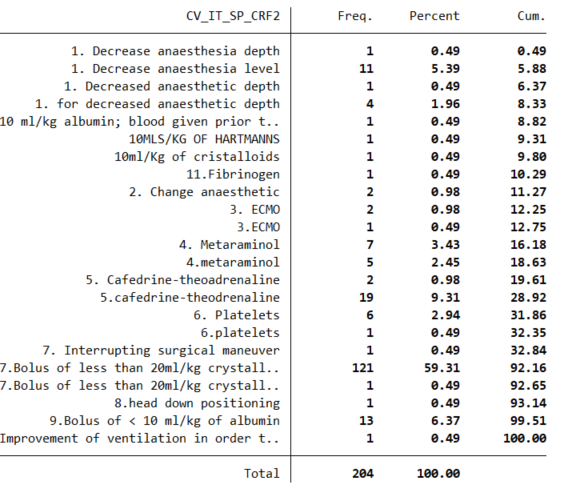

Supplement: Supplemental Digital Content [file ejanet-43-349-s001.docx]
